# *ATXN2* trinucleotide repeat length correlates with risk of ALS

**DOI:** 10.1016/j.neurobiolaging.2016.11.010

**Published:** 2017-03

**Authors:** William Sproviero, Aleksey Shatunov, Daniel Stahl, Maryam Shoai, Wouter van Rheenen, Ashley R. Jones, Safa Al-Sarraj, Peter M. Andersen, Nancy M. Bonini, Francesca L. Conforti, Philip Van Damme, Hussein Daoud, Maria Del Mar Amador, Isabella Fogh, Monica Forzan, Ben Gaastra, Cinzia Gellera, Aaron D. Gitler, John Hardy, Pietro Fratta, Vincenzo La Bella, Isabelle Le Ber, Tim Van Langenhove, Serena Lattante, Yi-Chung Lee, Andrea Malaspina, Vincent Meininger, Stéphanie Millecamps, Richard Orrell, Rosa Rademakers, Wim Robberecht, Guy Rouleau, Owen A. Ross, Francois Salachas, Katie Sidle, Bradley N. Smith, Bing-Wen Soong, Gianni Sorarù, Giovanni Stevanin, Edor Kabashi, Claire Troakes, Christine van Broeckhoven, Jan H. Veldink, Leonard H. van den Berg, Christopher E. Shaw, John F. Powell, Ammar Al-Chalabi

**Affiliations:** a Department of Basic and Clinical Neuroscience, King's College London, Maurice Wohl Clinical Neuroscience Institute, London, UK; b Department of Biostatistics, King's College London, Institute of Psychiatry, Psychology and Neuroscience, London, UK; c Department of Molecular Neuroscience, University College London (UCL) Institute of Neurology, London, UK; d Department of Neurology, Brain Center Rudolf Magnus Institute of Neuroscience, University Medical Centre Utrecht, Utrecht, the Netherlands; e Department of Clinical Neuropathology, King's College Hospital NHS Foundation Trust, London, UK; f Department of Pharmacology and Clinical Neuroscience, Umeå University, Umeå, Sweden; g Department of Biology, University of Pennsylvania, Philadelphia, PA, USA; h Institute of Neurological Sciences, National Research Council, Cosenza, Italy; i Neurology Department, University Hospitals Leuven, Leuven, Belgium; j Vesalius Research Center, VIB, Leuven, Belgium; k Disease (LIND), KU Leuven - University of Leuven, Leuven, Belgium; l Montreal Neurological Institute, McGill University, Montreal, Quebec, Canada; m Department of Nervous System Diseases, ALS Paris ALS Center for Rare Diseases, Groupe Hospitalier Pitié Salpêtrière, APHP, Paris, France; n Clinical Genetics Unit, Department of Woman and Child Health, University of Padova, Padova, Italy; o Fondazione IRCCS Istituto Neurologico Carlo Besta, Milan, Italy; p Department of Genetics, Stanford University School of Medicine, Stanford, CA, USA; q Sobell Department of Motor Neuroscience and Movement Disorders, University College London (UCL) Institute of Neurology, London, UK; r ALS Clinical Research Center, Bio. Ne. C., University of Palermo, Palermo, Italy; s Institut du Cerveau et de la Moelle épinière (ICM), Inserm U1127, CNRS UMR 7225, Sorbonne Universités, UPMC Univ Paris 06 UMRS1127, Paris, France; t AP-HP, Hôpitaux Universitaires La Pitié Salpêtrière-Charles Foix, Centre de Référence des Démences Rares, Departement de Neurologie, Paris, France; u Neurodegenerative Brain Diseases Group, Department of Molecular Genetics, VIB, Antwerp, Belgium; v Laboratory of Neurogenetics, Insititute Born-Bunge, University of Antwerp, Antwerp, Belgium; w Department of Neurology, Antwerp University Hospital, Edegem, Belgium; x Department of Neurology, Taipei Veterans General Hospital, Taipei, Taiwan; y Department of Neurology, National Yang-Ming University School of Medicine, Taipei, Taiwan; z Brain Research Center, National Yang-Ming University, Taipei, Taiwan; aa North-East London and Essex MND Care Centre - Neuroscience and Trauma Centre, Blizard, Institute of Cell and Molecular Medicine, Barts & the London School of Medicine & Dentistry, Barts Health NHS Trust, London, UK; bb Hôpital de la Pitié-Salpêtrière, institut de recherche translationnelle en neurosciences (A-ICM), Paris, France; cc Hôpital de la Pitié-Salpêtrière, réseau SLA IdF, Paris, France; dd Department of Clinical Neuroscience, University College London (UCL) Institute of Neurology, London, UK; ee Department of Neuroscience, Mayo Clinic, Jacksonville, FL, USA; ff Department of Neurosciences, University of Padova, Padova, Italy; gg Neurogenetics team, Ecole Pratique des Hautes Etudes, Paris, France

**Keywords:** ATXN2, SCA2, ALS, CAG, Expansion, Intermediate expansion, Trinucleotide repeat, Amyotrophic lateral sclerosis, Age of onset, Risk, Exponential risk, Triplet

## Abstract

We investigated a CAG trinucleotide repeat expansion in the *ATXN2* gene in amyotrophic lateral sclerosis (ALS). Two new case-control studies, a British dataset of 1474 ALS cases and 567 controls, and a Dutch dataset of 1328 ALS cases and 691 controls were analyzed. In addition, to increase power, we systematically searched PubMed for case-control studies published after 1 August 2010 that investigated the association between *ATXN2* intermediate repeats and ALS. We conducted a meta-analysis of the new and existing studies for the relative risks of *ATXN2* intermediate repeat alleles of between 24 and 34 CAG trinucleotide repeats and ALS. There was an overall increased risk of ALS for those carrying intermediate sized trinucleotide repeat alleles (odds ratio 3.06 [95% confidence interval 2.37–3.94]; *p* = 6 × 10^−18^), with an exponential relationship between repeat length and ALS risk for alleles of 29–32 repeats (R^2^ = 0.91, *p* = 0.0002). No relationship was seen for repeat length and age of onset or survival. In contrast to trinucleotide repeat diseases, intermediate *ATXN2* trinucleotide repeat expansion in ALS does not predict age of onset but does predict disease risk.

## 1 Introduction

Spinocerebellar ataxia type 2 is a trinucleotide repeat disease in which neurodegeneration is a consequence of expansion of a repeated CAG sequence in the *ATXN2* gene. All trinucleotide repeat diseases show neurological features and include Huntington's disease (Paulsen et al., 2014), the spinocerebellar ataxias (Tezenas du Montcel et al., 2014), Friedreich's ataxia (Koeppen, 2011), fragile X syndrome (Jin and Warren, 2000), myotonic dystrophy (Khoshbakht et al., 2014), and Kennedy's disease (Yang and Yamamoto, 2014) among others. The mechanism by which the repeated sequence causes disease remains unknown, but a frequently observed feature is a relationship between age of symptom onset, severity of phenotype, and repeat size, with larger repeats associated with younger onset and more severe disease (Nestor and Monckton, 2011).

Intriguingly, trinucleotide repeat expansion in the *ATXN2* gene is also a risk factor for amyotrophic lateral sclerosis (ALS), a neurodegenerative disease of upper and lower motor neurons, but this association is only seen for repeats of intermediate size, below the range usually associated with spinocerebellar ataxia (34 repeats or more) but above the normal range (Elden et al., 2010). Such pleiotropy is not seen in other trinucleotide repeat diseases and means that the usually observed relationship between repeat size, age of onset, and severity, might not be straightforward. Here, we investigate the size range defining ALS risk and test the relationship of phenotype with repeat size.

## 2 Methods

### 2.1 Unpublished case-control studies

A total of 1474 UK DNA samples of unrelated ALS patients (29 with an affected first degree relative) were collected from a consecutive clinical case series obtained from King's College Hospital (n = 116), from the Motor Neurone Disease Association DNA Biobank (n = 1051), and from Queen Mary University of London and UCL Institute of Neurology (n = 307). All patients were diagnosed as having definite or probable ALS according to the El Escorial criteria. The DNA samples of 567 neurologically normal controls, matched to patients for gender, age, and geographical region, were obtained from the MRC London Neurodegenerative Diseases Brain Bank, the Institute of Psychiatry, Psychology and Neuroscience (n = 68), from the National Institute for Health Research Mental Health Biomedical Research Centre and the Dementia Unit at South London and Maudsley NHS Foundation Trust and the Institute of Psychiatry, King's College London (n = 306), and from the Motor Neurone Disease Association (n = 193).

A second case-control population-based set was obtained in collaboration with the University Medical Center Utrecht, the Netherlands, with a total of 1328 unrelated ALS cases (23 with a family history in a first degree relative) and 691 neurologically normal controls, matched to patients for gender, age, and geographical region (Liberati et al., 2009). Samples used did not overlap with previous studies of *ATXN2* repeat size.

### 2.2 Standard protocol approvals, registrations, and patient consents

Informed consent was obtained from all included in the study. The study was approved by the Trent Research Ethics Committee 08/HO405/60 and by the Medical Ethics Review Board at the University Medical Center Utrecht 05_067/E.

### 2.3 Genetic analysis

The DNA samples of 1167 ALS cases and 567 controls were analyzed at the Institute of Psychiatry, Psychology and Neuroscience, King's College London, and DNA samples of 307 ALS cases underwent analysis at the Institute of Neurology, UCL. The *ATXN2* CAG trinucleotide repeat region was amplified according to a previously published PCR protocol (Pulst et al., 1996). PCR products at King's College London were run on an Applied Biosystems 3130xl Genetic Analyzer, and those at UCL on an Applied Biosystems 3730xl Genetic Analyzer. PCR fragments were analyzed using GeneMapper V 4.0 software (Applied Biosystems) to determine CAG trinucleotide repeat size. Electropherogram peaks were sized using GeneScanTM 500 LIZ as reference dye labeled standard. Sequenced samples of known CAG trinucleotide repeat size were used as internal controls for both PCR and GeneScan analysis. PCR products of cases and controls with more than 26 repeats were regenotyped to validate the obtained results.

The DNA samples of 1328 Dutch ALS cases and 691 controls underwent *ATXN2* CAG trinucleotide repeat amplification according to a previously reported PCR protocol (Van Damme et al., 2011). PCR products were analyzed using an Applied Biosystems 3130xl Genetic Analyzer. PCR fragments were analyzed using GeneMapper V 4.0 software (Applied Biosystems) to determine CAG trinucleotide repeat size. Electropherogram peaks were sized using GeneScanTM 500 LIZ as reference dye labeled standard. Sequenced samples of known CAG trinucleotide repeat size were used as internal controls for both PCR and GeneScan analysis. PCR products of cases and controls with more than 26 repeats were regenotyped to validate the obtained results.

Samples were also genotyped for C9orf72 expansion as described previously (See Supplementary Material).

### 2.4 Inclusion criteria for published studies

Systematic review and meta-analysis were conducted in accordance with the PRISMA (Huisman et al., 2011; Preferred Reporting Items for Systematic reviews and Meta-Analyses) group guidelines and Cochrane Collaboration. The types of studies included were case-control studies designed to evaluate the minimum number of CAG repeats in the *ATXN2* gene conferring risk for ALS. Series of cases and descriptive reports were excluded from study selection. Repeats of size 23 or less were regarded as normal given their high control frequency in several populations (Laffita-Mesa et al., 2012).

### 2.5 Study design, data extraction, and control of bias

This was not an interventional study and therefore was not randomized or blinded. Study selection was restricted to case-control studies published after 1 August 2010, the date of the first reported association between *ATXN2* variation and ALS. The exact frequencies of each allele with 24 repeats or greater, and the pooled counts of alleles frequencies with less than 24 repeats were extracted for both cases and controls from published papers. ALS diagnostic criteria, control recruitment information, and case-control matching for age and geographical region were extracted to ensure comparability between studies. Where *ATXN2* allele frequencies or information about control selection and case-control age matching were incomplete, study authors were contacted. Data extraction was performed in duplicate by 2 independent investigators (William Sproviero, Aleksey Shatunov). The new case-control studies from UK and Dutch populations were included in the analysis. Bias in individual studies was evaluated using the Newcastle-Ottawa Scale questionnaire for Quality Assessment of Nonrandomized Studies (Stang, 2010). The questionnaire contains 8 items subdivided into 3 categories (selection, comparability, and exposure), with a maximum overall score of 8. Studies with total score equal or greater than 6 were considered at low risk of bias.

### 2.6 Statistical methods

Relative risks (RRs) were approximated by the odds ratio, generated with corresponding 95% confidence intervals (CIs), by meta-analysis using a Cochran-Mantel-Haenszel chi-square test comparing the case-control counts for a specific allele with the pooled counts for alleles of 23 repeats or fewer across the different published and unpublished studies. RR was then estimated comparing pooled counts of risk alleles with counts of alleles of 23 repeats or fewer. Where a cell contained zero observations, a continuity correction of 0.5 was applied. The sample size was considered adequate to measure the effect size since each individual study in the meta-analysis measured the effect, and the addition of further samples would increase power further. We assumed that all studies were estimating the same common effect and estimates varied only because of chance differences in sampling patients. To assess our assumption, heterogeneity between studies was estimated using the I^2^ statistic (% of variability due to between-study heterogeneity) and Cochrane's Q-test of heterogeneity. I^2^ > 50% or *p* < 0.05 for the Q-test were taken as indicative of significant heterogeneity. We used a fixed effects model following the assumption that all studies had a common genetic effect and that specific findings of each study were due to random sampling. However, to control for any possible difference across studies, the fixed effect model RR estimates at each threshold were compared with RR estimates assessed using a random effects model. A sensitivity analysis, leaving out one study at a time, was performed to test the robustness of the meta-analysis and assess the influence of individual studies on the overall result for each allele. Possible sources of heterogeneity across studies were explored using subgroup analysis using source of the control group (population based vs. nonpopulation based) and geographic location (China, Europe, Turkey, USA) as covariates. Meta-regression was used to further investigate differences between population-based and nonpopulation-based subgroups. Funnel plots were generated for each intermediate repeat allele to analyze the intervention effect from individual studies against study size. A resulting *p* < 0.05 was considered as indicative of the presence of small-study effects. Correlation between age at onset and the CAG trinucleotide repeat length of risk alleles was tested in the new British and Dutch ALS cases, and in published data sets for which age at onset data were available. Two-tailed Fisher exact tests were used to test for differences in demographic and clinical characteristics of patients by *ATXN2* repeat size. ANOVA was used to compare ages at onset for different repeat sizes, as well as by using SNPs rs695871 and rs695872, previously shown to associate with age of onset. Kaplan-Meier survival analysis and a log-rank test were used to compare survival time between groups. We compared the fit of an exponential model with the fit of a linear model using Akaike information criteria and Bayesian information criteria. Comparison of model values for either measure can be used to assess fit provided the values differ by more than 10. The model with the larger value has less support (Burnham and Anderson, 2002, Raftery, 1995). Meta-analyses were performed using STATA version 12.0 (Stata Corp, College Station, TX, USA). Chi-square tests, ANOVA, and Kaplan-Meier survival analysis were performed using SPSS statistical package version 22 (IBM, Chicago, IL, USA). R language (http://www.R-project.org) was used to test the hypothesis that the relationship between *ATXN2* CAG repeat length and ALS risk fitted an exponential model.

## 3 Results

**Fig. 1:**
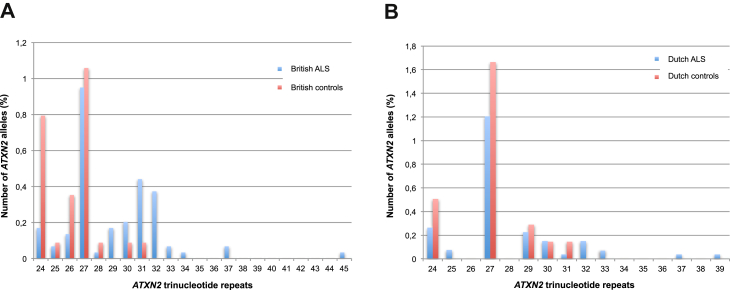
Distribution of *ATXN2* alleles with trinucleotide repeat size 24 or more in the (A) British and (B) Dutch datasets. (A) The British dataset included 1474 ALS individuals and 574 controls. There were 2867 alleles of size 23 or less in cases and 1105 in controls. (B) The Dutch dataset included 1328 ALS individuals and 691 controls. There were 2596 alleles of size 23 or less in cases and 1344 in controls.

*ATXN2* trinucleotide CAG repeats were analyzed in 1474 ALS cases and 567 neurologically normal controls from the UK and in 1328 ALS cases and 691 neurologically normal controls from the Netherlands. The distribution of allele frequencies is shown in Fig. 1.

All cases were tested for the copresence of *ATXN2* intermediate expansions and *C9orf72* expansion. Six patients (2 UK, 4 Dutch) had intermediate *ATXN2* expansion and pathological expansion of *C9orf72*. Exclusion of these patients from analyses did not change the overall findings.

**Fig. 2:**
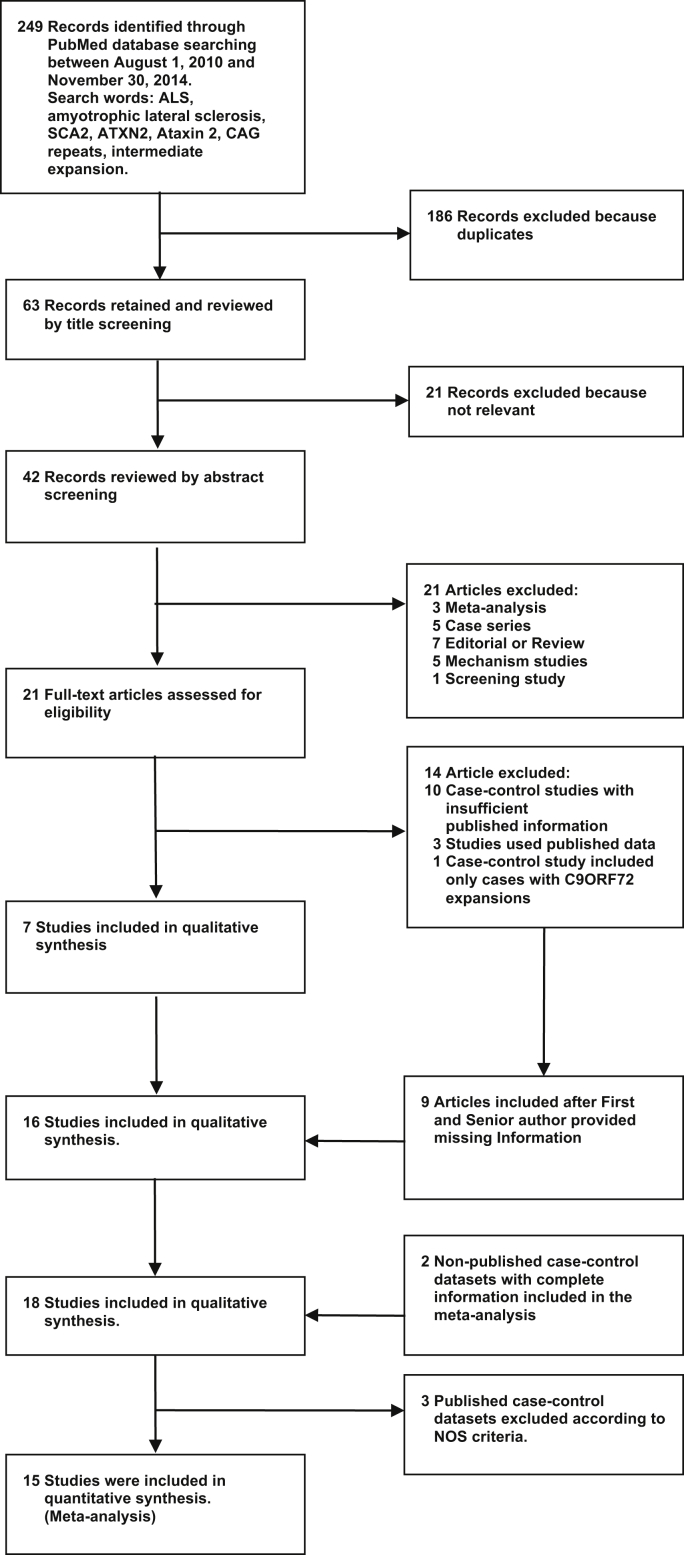
Flow chart of study selection according to the PRISMA protocol. Thirteen previously published studies were selected for analysis, 7 from Europe (Conforti et al., 2012, Corrado et al., 2011, Daoud et al., 2011, Gispert et al., 2012, Lattante et al., 2014, Van Damme et al., 2011, Van Langenhove et al., 2012), 3 studies of Han Chinese (2 from China [Liu et al., 2013, Lu et al., 2015] and 1 from Taiwan [Soong et al., 2014]), 1 from Turkey (Lahut et al., 2012), and 2 studies from the USA (Elden et al., 2010, Ross et al., 2011).

Based on literature searches (Fig. 2), we identified all known studies examining *ATXN2* repeat expansion in ALS, contacting authors for raw data where necessary, and including studies for analysis based on strict criteria (Supplementary Table 1). Studies passing inclusion criteria (Conforti et al., 2012, Corrado et al., 2011, Daoud et al., 2011, Elden et al., 2010, Gellera et al., 2012, Gispert et al., 2012, Lahut et al., 2012, Lattante et al., 2014, Lee et al., 2011, Liu et al., 2013, Lu et al., 2015, Ross et al., 2011, Soong et al., 2014, Sorarù et al., 2011, Van Damme et al., 2011, Van Langenhove et al., 2012) and the 2 novel datasets were used. One Chinese dataset (Chen et al., 2011) was excluded because the authors were unable to provide information on the control group. We excluded studies that might show bias according to the Newcastle-Ottawa Scale criteria (Supplementary Table 2), leaving a total of 15 studies for meta-analysis, comprising 10,888 cases and 15,463 controls (Supplementary Fig. 1). The allele counts of pooled alleles <24 repeats and of each allele with 24 trinucleotide repeats or greater are reported in Supplementary Table 3. No evidence of small-study effect was found in any of the primary analysis studies (data not shown), although we acknowledge that the Funnel plots are not independent of each other.

We first established the definitive size range ascribing risk for ALS, investigating each allele from 24 repeats to 34 repeats by using a fixed effects approach (Supplementary Table 4). The exclusion of the study published by Liu et al. (2013) from analysis of the RR conferred by the 30 repeat allele lowered the initial significant heterogeneity (I^2^ = 48.8%, heterogeneity *p* = 0.02) to an overall I^2^ value of 0% (heterogeneity *p* = 0.51) but did not influence the overall RR estimate. No study exclusion could explain the significant heterogeneity present in the analysis of the 24 repeat allele. No other studies had any effect on heterogeneity or RR estimates, including 2 studies that used young controls below the age of risk (Corrado et al., 2011, Elden et al., 2010), and there was no effect of ancestral background of the population studied. Using a random effects model did not change the findings (Supplementary Table 5).

**Fig. 3:**
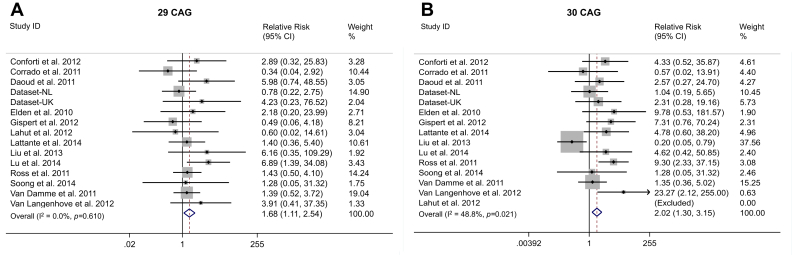

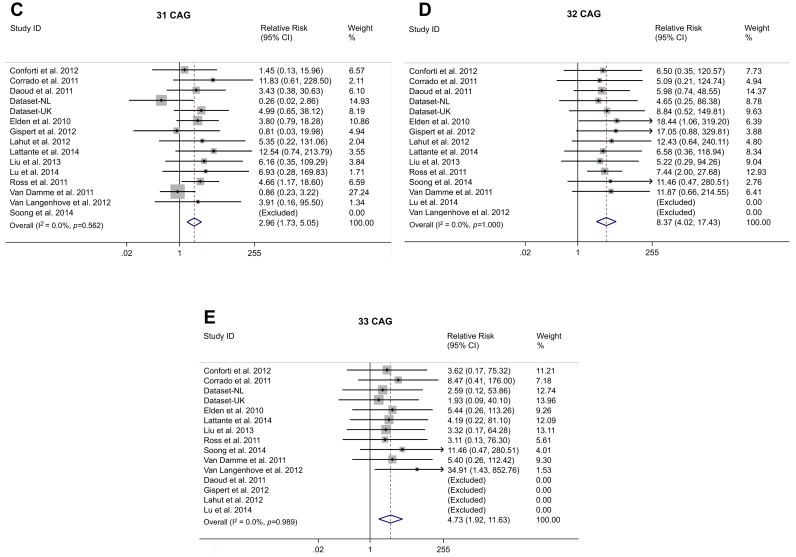
Forest plots of individual alleles between 29 and 33 repeats. Primary analysis of the relative risk of ALS was conducted using low risk-bias case-control studies. UK = new UK data, NL = new Dutch data. Relative risk (RR) was estimated using a fixed effects approach and by comparing individual counts of *ATXN2* alleles with the pooled count of alleles with ≤23 repeats as baseline. (A) *ATXN2* allele of 29 repeats, RR = 1.68 (1.11, 2.54). No heterogeneity was observed (*p*-value of heterogeneity >0.05). (B) *ATXN2* allele of 30 repeats, RR = 2.02 (1.30, 3.15). One of 15 studies was excluded for absence of carriers of allele 30, both in cases and controls. Significant heterogeneity was observed (*p*-value of heterogeneity <0.05). (C) *ATXN2* allele of 31 repeats, RR = 2.96 (1.73, 5.05). One of 15 studies was excluded for absence of carriers of allele 31, both in cases and controls. No heterogeneity was observed (*p*-value of heterogeneity >0.05). (D) *ATXN2* allele of 32 repeats, RR = 8.37 (4.02, 17.43). Two of 15 studies were excluded for absence of carriers of allele 32, both in cases and controls. No heterogeneity was observed (*p*-value of heterogeneity >0.05). (E) *ATXN2* allele of 33 repeats, RR = 4.73 (1.92, 11.63). No heterogeneity was observed (*p*-value of heterogeneity >0.05). Abbreviation: ALS, amyotrophic lateral sclerosis.

**Fig. 4:**
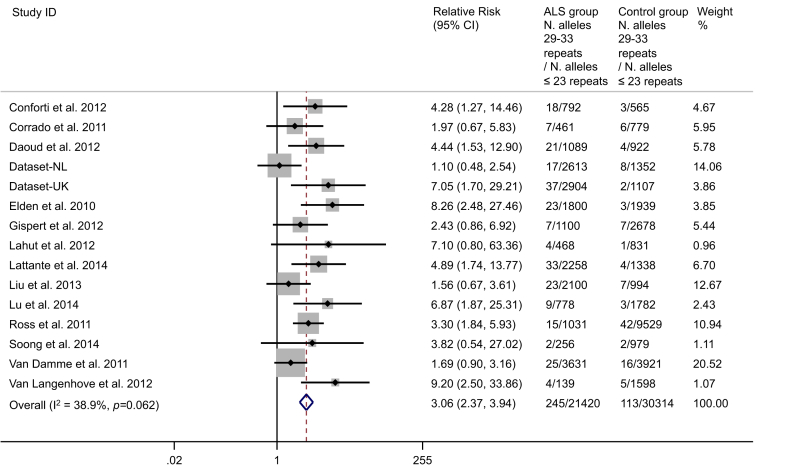
Forest plot of the relative risk of ALS for *ATXN2* alleles with 29–33 trinucleotide repeats. UK = new UK data, NL = new Dutch data. Fifteen studies at low risk of bias were included. Fixed effects methods were used to estimate the relative risk. No heterogeneity was observed (*p*-value of heterogeneity >0.05). Abbreviation: ALS, amyotrophic lateral sclerosis.

We found that alleles with 29–33 repeats were associated with ALS (Fig. 3). A meta-analysis of the pooled counts of the risk alleles showed a RR of ALS of 3.06, 95% CI, 2.37–3.94, *p* = 6 × 10^−18^ (Fig. 4). We performed a sensitivity analysis, reintroducing the 3 studies excluded for risk of bias, which did not affect the results (data not shown).

**Fig. 5:**
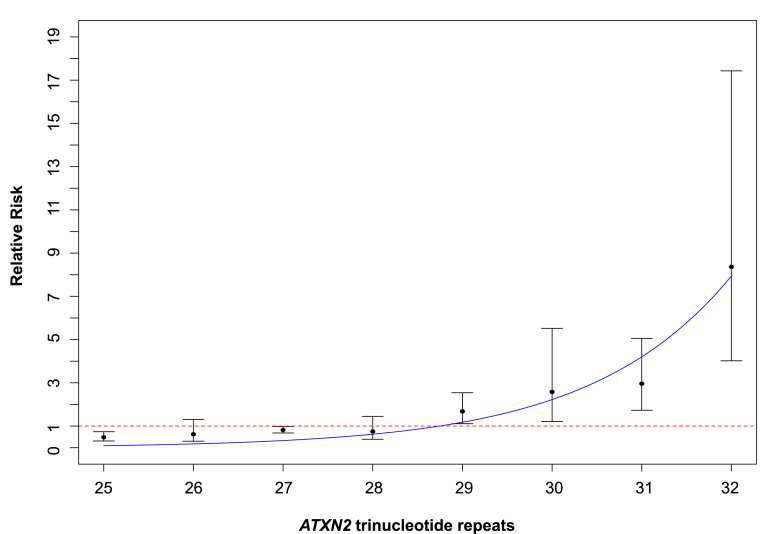
Plot of the relative risk for each *ATXN2* allele (25–32 repeats). The distribution of the relative risk estimates of alleles of between 25 and 32 CAG trinucleotide repeats obtained from the 15 low bias studies fitted an exponential curve well, showing an exponential growth in relative risk, surpassing the threshold for significant association for alleles of size 29–32 (*R*^2^ = 0.91 [95% CI 0.82, 0.99], *p* = 0.0002). The relative risk estimate of the 24 repeat allele was excluded because of a large unidentified heterogeneity across studies. Including this allele, however, did not significantly change the curve fit. Black bars indicate the 95% CI of the relative risk estimates. The red line indicates no effect. Abbreviation: CI, confidence interval.

Investigating the effect size of each allele, we found that the risk increased exponentially with length for alleles of 29–32 repeats (*R*^2^ = 0.91 [95% CI 0.82, 0.99], *p* = 0.0002; Fig. 5), only dropping off at the boundary for risk of spinocerebellar ataxia type 2, at 33 repeats. This is surprising and has not been reported for any trinucleotide repeat disease. The goodness-of-fit of the exponential model was compared with the fit of a linear model. The exponential model gave a better fit based on Akaike information criteria and Bayesian information criteria criteria (Supplementary Table 6).

Next, we tested the relationship between repeat length and age of ALS onset in the 4 different populations for which data were available. In keeping with previous findings, and in contrast to trinucleotide repeat diseases, we found no evidence for such a relationship (UK [n = 17] age at onset-repeat length regression, *p* = 0.90; the Netherlands [n = 37] age at onset-repeat length regression, *p* = 0.08; Belgium [Van Damme et al., 2011; n = 25] age at onset-repeat length regression, *p* = 0.83; France [Lattante et al., 2014; n = 33] age at onset-repeat length regression, *p* = 0.49; Flanders-Belgian [Van Langenhove et al., 2012; n = 4] age at onset-repeat length regression, *p* = 0.60; overall age at onset-repeat length regression, *p* = 0.14). Nor were there any associations when SNPs rs695871 and rs695872, previously shown to associate with the age of onset, were tested.

We also assessed differences in demographic and clinical characteristics between patients with CAG repeats <29 and patients with CAG repeats ≥29 in both British and Dutch cohorts. No significant difference was found in gender, age at onset, or site of onset (Supplementary Table 7). No significant difference in survival was detected by Kaplan-Meier analysis in either the British (*p*-value = 0.87) or Dutch (*p*-value = 0.31) cohorts.

## 4 Discussion

We have found the risk range for *ATXN2* trinucleotide repeat alleles in ALS is 29–33. An unexpected and important finding is that the risk of ALS increases exponentially with allele repeat size until the border with spinocerebellar ataxia risk, even though the age of onset does not change. This may appear surprising but is entirely consistent with current hypotheses of ALS causation, in which the odds ratio conferred by genetic variants and age of onset of first symptoms are not correlated, even within the same family (e.g., *TARDBP*, *FUS*; Abel et al., 2012, Al-Chalabi and Hardiman, 2013, van Rheenen et al., 2016). Our confidence in the finding of an exponential increase in risk is high because this is the largest study of *ATXN2* alleles and ALS, our findings overall are consistent with previous studies, and 4 different European populations gave identical results for the relationship of age of onset with trinucleotide allele repeat size. Furthermore, an exponential fit is strongly supported statistically over a linear change in risk. The fact the risk drops off at 33 repeats can be interpreted as a dilution in the case ascertainment, since a large proportion of people with 33 repeats would develop spinocerebellar ataxia (Fernandez et al., 2000) rather than ALS, but we have ascertained on disease state rather than repeat size. To ascertain repeat size, we used a method previously reported to include unrelated PCR products in the critical range of the gel (Pulst et al., 1996). To overcome this limitation, we genotyped all samples twice.

The trinucleotide repeat size range we found associated with risk of ALS overlapped with but was larger than previously reported ranges, (probably because the increased sample size improved our statistical power), and has implications for genetic counseling of individuals carrying intermediate size repeats. Two published meta-analyses, one of 12 and another of 13 studies, reported significant association with ALS for pooled analyses of *ATXN2* alleles greater than 30 trinucleotide repeats, a finding that remained significant when restricted to alleles sized between 30 and 33 repeats (Laffita-Mesa et al., 2012, Wang et al., 2014). Another meta-analysis of 9 studies found that individual *ATXN2* allele frequencies of 31, 32, and 33 repeats were significantly higher in ALS cases than in controls (Neuenschwander et al., 2014).

It has been suggested that shorter intermediate repeats may be protective for ALS (Neuenschwander et al., 2014). Our data are consistent with this possibility, and indeed for alleles of 27 repeats, show a significant protective effect (Fig. 5, Supplementary Table 4). Previous studies of the *ATXN2* repeat length in ALS have not shown a relationship with age of onset (Laffita-Mesa et al., 2012, Neuenschwander et al., 2014, Wang et al., 2014). In this study, 5 populations comprising 5703 cases of the 10,888 individuals studied had age of onset data available. Despite this limitation in numbers, there is unlikely to be a major effect of allele size on the age of onset for ALS. A recent study reported a modifier effect of *ATXN2* intermediate length repeats on ALS survival for those with 31 or more repeats (Chiò et al., 2015). We did not replicate this finding.

The mechanism by which expanded trinucleotide repeats cause disease does not appear to be the same for all such diseases, even though all trinucleotide repeat expansions result in neurological dysfunction. In some cases, there is a loss of function, for example through hypermethylation (Jin and Warren, 2000), whereas in others, there is a toxic gain of function, for example through aggregation following protein misfolding (Kayatekin et al., 2014). For toxic products of affected genes, larger expansions are likely to result in increased toxicity, and since the products are present from birth, an earlier age of onset or more severe phenotype is the likely outcome of larger repeat sizes. Here, we can add a third outcome of larger repeats: that the risk of disease increases. This can be explained within the recently proposed multistep model of ALS if the toxic effect of intermediate expansions is one of the steps required for ALS to develop, and the toxic effect shows a correlation with repeat size (Al-Chalabi et al., 2014). It also allows for the possibility of oligogenic inheritance, where multiple genetic risk factors act in concert to cause ALS. The mechanism by which this might happen without a concomitant reduction in age of onset remains to be determined. A possible explanation lies in the existence of CAA interruptions to the CAG trinucleotide repeat sequence. A weakness of this study is that such interruptions would not be detectable using our assay but would merely appear as additional CAG repeats. Up to 3 such interruptions have been observed in the *ATXN2* trinucleotide repeat by direct sequencing but become less likely as the length of the repeat increases. When CAA interruptions occur in repeats of size 33–40, the phenotype is usually of a dopa-responsive Parkinsonism (Kim et al., 2007). One mechanism for risk associated with CAG repeats is through RNA toxicity, and CAA interruptions specifically alter RNA secondary structure. The formation of a stable RNA hairpin structure could be associated with the position and number of CAA triplets along the CAG expansion leading to an RNA toxic gain of function (Yu et al., 2011). Thus the interruptions can modify phenotype, and an interaction between repeat length and CAA interruption could underlie ALS risk and the lack of effect on the age of onset. Furthermore, although both Dutch and UK cohorts had individuals with more than 33 repeats, they did not have spinocerebellar ataxia, a finding which might be related to CAA interruptions.

## 5 Conclusion

Our study increases the breadth of known effects of trinucleotide repeat expansion size, adding disease risk to the existing correlations with age of onset and disease severity. Thus, the main finding presented here is that trinucleotide repeat expansion in the *ATXN2* gene in the size range exclusively for ALS risk represents an exponentially increasing risk for each additional repeat.

## Disclosure statement

The authors have no conflicts of interest to disclose.
