# Supplementary material for: ATXN2 trinucleotide repeat length correlates with risk of ALS
Source: Neurobiol Aging. 2017 Mar;51:178.e1–9. doi: 10.1016/j.neurobiolaging.2016.11.010 (PMC5302215; doi:10.1016/j.neurobiolaging.2016.11.010)
Supplement: Supplementary Data [file mmc1.docx]

**Supplementary Data**

**Supplementary Methods**

*C9orf72 Repeat-Prime PCR*

A repeat-primed PCR reaction was performed in order to perform a qualitative assessment of the presence of an expanded (GGGGCC)n hexanucleotide repeat in *C9ORF72.* The PCR method used was previously published by DeJesus-Hernandez M et al 2011. PCR products were analyzed on an ABI3130xl DNA Analyzer and visualized using GeneMapper software. To avoid false-negative calls, we genotyped all samples at least twice.

*SNPs rs695871 and rs695872*

Genetic information regarding two SNPs (rs695871; rs695872), previously reported to be associated with age at onset (Yu et al. 2011), were obtained from genotyped data previously published (van Rheenen et al 2016).

**Supplementary Results**

*Genetic Analyses for UK cases*

There were 78 UK ALS patients with *C9orf72* expansion, 76 with *ATXN2* alleles of size < 24 CAG repeats, two with intermediate sized repeats.

*Genetic Analysis for Dutch cases*

There were 92 Dutch ALS patients with *C9orf72* expansion, 88 with *ATXN2* alleles of size < 24 CAG repeats, four with intermediate sized repeats.

**Supplementary Table 1: Summary of the published studies included in the systematic review.**

| **Study** | **Geographical location** | **ALS Diagnosis Criteria** | **Cases and controls age matched** | **Cases and controls geographically matched** | **Controls without neurodegenerative diseases** | **Controls Selection** | **Laffita-Mesa et al. 2013** | **Wang et al. 2014** | **Neuenschwander et al. 2014** |
| --- | --- | --- | --- | --- | --- | --- | --- | --- | --- |
|  |  |  |  |  |  |  |  |  |  |
| Conforti et al. 2012† | European (Italian) | El-Escorial | Yes | Yes | Yes | Hospital controls | Present | Present | - |
| Corrado et al. 2011 | European (Italian) | El-Escorial | No | Yes | Yes | Blood donors; Hospital and University staff. | Present | Present | Present |
| Daoud et al. 2012† | Eurpean (French and French-Canadian) | El-Escorial | Yes | Yes | Yes | Patient spouses | Present | Present | Present |
| Elden et al. 2010 | North American (Caucasian origin) | El-Escorial | No | Yes | Yes | Hospital controls | Present | Present | Present |
|  |  |  |  |  |  |  |  |  |  |
| Gellera et al. 2012† | European (Italian) | El-Escorial | NA | Yes | NA | Blood donors | Present | Present | Present |
| Gispert et al. 2012 | European (German-Swiss) | European guidelines | Yes | Yes | Yes | Population-Based | Present | Present | Present |
| Lahut et al. 2012 | Asian (Turkish) | El-Escorial | Yes | Yes | Yes | Hospital controls | - | Present | Present |
| Lattante et al. 2014† | Eurpean (French) | El-Escorial | Yes | Yes | Yes | Patient spouses | - | - | - |
| Lee et al. 2011 | European (Swedish, German, Swiss, Finland, Norwegian, Danish, Portuguese, Iceland) | European guidelines | Yes | No | NA | Patient spouses; Blood donors. | Present | Present | - |
| Liu et al. 2013† | Chinese (mainland) | El-Escorial | Yes | Yes | Yes | Blood donors | - | Present | Present |
| Lu et al. 2014 | Chinese (mainland) | El-Escorial | Yes | Yes | Yes | Population-Based | - | - | - |
| Ross et al. 2011† | North American (Caucasian origin) | El-Escorial | Yes | Yes | Yes | Hospital controls | Present | Present | - |
| Soong et al. 2014† | Chinese (Taiwanese) | El-Escorial | Yes | Yes | Yes | Population-Based | - | - | - |
| Sorarù et al. 2011 | European (Italian) | El-Escorial | NA | Yes | No | Hospital controls; healthy relatives of hospitalized patients; ataxic patients. | Present | Present | - |
| Van Damme et al. 2011† | European (Belgian-Dutch) | El-Escorial | Yes | Yes | Yes | Population-Based | Present | Present | Present |
| Van Langenhove et al. 2012† | European (Flanders-Belgian) | El-Escorial | Yes | Yes | Yes | Population-Based | Present | - | Present |
| † = Information provided by contacted Authors. NA= Not Available. "-" =absent. | | | |  |  |  |  |  |  |

Two meta-analyses (Laffita-Mesa et al. 2013; Wang et al. 2014) included an article published by Chen et al. 2011 that was excluded in the present study by the criteria described in the Methods.

**Supplementary Table 2. Newcastle-Ottawa Scale (NOS) quality assessment questionnaire of nonrandomized studies.**

| **Study** | **Selection** |  |  |  | **Comparability** | **Exposure** |  |  | **Total Scores** |
| --- | --- | --- | --- | --- | --- | --- | --- | --- | --- |
|  | **Case Definition**  **Adequate** | **Representativeness**  **of the cases** | **Selection of**  **Controls** | **Definition of**  **Controls** | **Comparability of**  **cases and controls**  **(age matching)** | **Ascertainment**  **of exposure** | **Same method of**  **Ascertainment**  **for cases** | **Non-Response**  **value** |  |
| Conforti et al. 2012 | ★ | ★ | - | ★ | ★ | ★ | ★ | ★ | 7 |
| Corrado et al. 2011 | ★ | ★ | - | ★ | - | ★ | ★ | ★ | 6 |
| Daoud et al. 2012 | ★ | ★ | - | ★ | ★ | ★ | ★ | ★ | 7 |
| Dataset-NL ^*^ | ★ | ★ | ★ | ★ | ★ | ★ | ★ | ★ | 8 |
| Dataset-UK ^*^ | ★ | ★ | - | ★ | ★ | ★ | ★ | ★ | 7 |
| Elden et al. 2010 | ★ | ★ | - | ★ | - | ★ | ★ | ★ | 6 |
| Gellera et al. 2012 | ★ | ★ | - | - | - | - | - | ★ | 3 |
| Gispert et al. 2012 | ★ | ★ | ★ | ★ | ★ | ★ | ★ | ★ | 8 |
| Lahut et al. 2012 | ★ | ★ | - | ★ | ★ | ★ | ★ | ★ | 7 |
| Lattante et al. 2014 | ★ | ★ | - | ★ | ★ | ★ | ★ | ★ | 7 |
| Lee et al. 2011 | ★ | ★ | - | - | ★ | - | - | ★ | 4 |
| Liu et al. 2013 | ★ | ★ | - | ★ | ★ | ★ | ★ | ★ | 7 |
| Lu et al. 2014 | ★ | ★ | ★ | ★ | ★ | ★ | ★ | ★ | 8 |
| Ross et al. 2011 | ★ | ★ | - | ★ | ★ | ★ | ★ | ★ | 7 |
| Soong et al. 2014 | ★ | ★ | ★ | ★ | ★ | ★ | ★ | ★ | 8 |
| Sorarù et al. 2011 | ★ | ★ | - | - | - | - | - | ★ | 3 |
| Van Damme et al. 2011 | ★ | ★ | ★ | ★ | ★ | ★ | ★ | ★ | 8 |
| Van Langenhove et al. 2012 | ★ | ★ | ★ | ★ | ★ | ★ | ★ | ★ | 8 |
|  |  |  |  |  |  |  |  |  |  |
| * Unpublished datasets |  |  |  |  |  |  |  |  |  |

The table includes 16 published studies and two unpublished datasets. Chen et al. 2011 was excluded according to the criteria for published studies described in the “Methods” section and is therefore not reported in the table. In three studies (Gellera et al. 2012; Lee et al. 2011; Sorarù et al. 2011) the NOS score was < 5, suggesting bias. In fifteen studies the NOS score was between 6 and 8 showing a low risk of bias. Black stars indicate low risk of bias. Dashes indicate high risk of bias.

**Supplementary Table 3. *ATXN2* allele counts of all case-control cohorts included in the study.**

| **Study** | **Individuals** | **≤ 23** | **24** | **25** | **26** | **27** | **28** | **29** | **30** | **31** | **32** | **33** | **34** | **35** | **36** | **37** | **39** | **45** |
| --- | --- | --- | --- | --- | --- | --- | --- | --- | --- | --- | --- | --- | --- | --- | --- | --- | --- | --- |
| Conforti et al. 2012 | ALS cases | 773 | 3 | 4 | 0 | 6 | 0 | 4 | 6 | 2 | 4 | 2 | 1 | 1 | 2 | 0 | 0 | 0 |
|  | Controls | 562 | 0 | 10 | 0 | 11 | 0 | 1 | 1 | 1 | 0 | 0 | 0 | 0 | 0 | 0 | 0 | 0 |
| Corrado et al. 2011 | ALS cases | 454 | 1 | 0 | 0 | 1 | 0 | 1 | 0 | 3 | 1 | 2 | 0 | 0 | 0 | 1 | 0 | 0 |
|  | Controls | 773 | 3 | 0 | 0 | 7 | 1 | 5 | 1 | 0 | 0 | 0 | 0 | 0 | 0 | 0 | 0 | 0 |
| Daoud et al. 2012 | ALS cases | 1064 | 8 | 1 | 0 | 13 | 1 | 6 | 3 | 5 | 6 | 1 | 0 | 2 | 1 | 1 | 0 | 0 |
|  | Controls | 918 | 3 | 2 | 0 | 15 | 0 | 1 | 1 | 1 | 1 | 0 | 0 | 0 | 0 | 0 | 0 | 0 |
| Dataset-NL* | ALS cases | 2596 | 7 | 2 | 0 | 32 | 0 | 6 | 4 | 1 | 4 | 2 | 0 | 0 | 1 | 0 | 1 | 0 |
|  | Controls | 1344 | 7 | 0 | 0 | 23 | 0 | 4 | 2 | 2 | 0 | 0 | 0 | 0 | 0 | 0 | 0 | 0 |
| Dataset-UK* | ALS cases | 2867 | 5 | 2 | 4 | 28 | 1 | 5 | 6 | 13 | 11 | 2 | 1 | 0 | 0 | 2 | 0 | 1 |
|  | Controls | 1105 | 9 | 1 | 4 | 12 | 1 | 0 | 1 | 1 | 0 | 0 | 0 | 0 | 0 | 0 | 0 | 0 |
| Elden et al. 2010 | ALS cases | 1777 | 6 | 1 | 0 | 22 | 1 | 2 | 4 | 7 | 8 | 2 | 0 | 0 | 0 | 0 | 0 | 0 |
|  | Controls | 1936 | 4 | 4 | 2 | 11 | 0 | 1 | 0 | 2 | 0 | 0 | 0 | 0 | 0 | 0 | 0 | 0 |
| Gellera et al. 2012 | ALS cases | 1280 | 3 | 1 | 0 | 10 | 0 | 4 | 3 | 7 | 6 | 2 | 0 | 0 | 0 | 0 | 0 | 0 |
|  | Controls | 1076 | 3 | 2 | 1 | 14 | 1 | 3 | 1 | 1 | 0 | 0 | 0 | 0 | 0 | 0 | 0 | 0 |
| Gispert et al. 2012 | ALS cases | 1093 | 1 | 1 | 0 | 15 | 0 | 1 | 3 | 0 | 3 | 0 | 0 | 1 | 0 | 0 | 0 | 0 |
|  | Controls | 2671 | 9 | 1 | 0 | 48 | 1 | 5 | 1 | 1 | 0 | 0 | 1 | 0 | 0 | 0 | 0 | 0 |
| Lahut et al. 2012 | ALS cases | 464 | 3 | 0 | 0 | 1 | 0 | 0 | 0 | 1 | 3 | 0 | 0 | 0 | 0 | 0 | 0 | 0 |
|  | Controls | 830 | 4 | 1 | 0 | 3 | 1 | 1 | 0 | 0 | 0 | 0 | 0 | 0 | 0 | 0 | 0 | 0 |
| Lattante et al. 2014 | ALS cases | 2225 | 5 | 1 | 0 | 18 | 1 | 7 | 8 | 10 | 5 | 3 | 1 | 1 | 1 | 1 | 1 | 0 |
|  | Controls | 1334 | 5 | 1 | 1 | 17 | 2 | 3 | 1 | 0 | 0 | 0 | 0 | 0 | 0 | 0 | 0 | 0 |
| Lee et al. 2011 | ALS cases | 2537 | 3 | 1 | 1 | 26 | 1 | 5 | 0 | 6 | 3 | 4 | 0 | 1 | 0 | 0 | 0 | 0 |
|  | Controls | 1328 | 6 | 2 | 2 | 13 | 3 | 2 | 2 | 0 | 0 | 0 | 0 | 0 | 0 | 0 | 0 | 0 |
| Liu et al. 2013 | ALS cases | 2077 | 11 | 10 | 4 | 4 | 2 | 6 | 3 | 6 | 5 | 3 | 2 | 1 | 0 | 0 | 0 | 0 |
|  | Controls | 987 | 0 | 11 | 1 | 2 | 4 | 0 | 7 | 0 | 0 | 0 | 0 | 0 | 0 | 0 | 0 | 0 |
| Lu et al. 2014 | ALS cases | 769 | 6 | 1 | 1 | 0 | 2 | 6 | 2 | 1 | 0 | 0 | 0 | 0 | 0 | 0 | 0 | 0 |
|  | Controls | 1779 | 1 | 10 | 4 | 1 | 2 | 2 | 1 | 0 | 0 | 0 | 0 | 0 | 0 | 0 | 0 | 0 |
| Ross et al. 2011 | ALS cases | 1016 | 8 | 2 | 1 | 20 | 1 | 4 | 4 | 3 | 4 | 0 | 0 | 0 | 1 | 0 | 0 | 0 |
|  | Controls | 9487 | 44 | 13 | 8 | 156 | 4 | 26 | 4 | 6 | 5 | 1 | 0 | 0 | 0 | 0 | 0 | 0 |
| Soong et al. 2014 | ALS cases | 254 | 1 | 2 | 0 | 0 | 1 | 0 | 0 | 0 | 1 | 1 | 0 | 0 | 0 | 0 | 0 | 0 |
|  | Controls | 977 | 4 | 8 | 3 | 1 | 5 | 1 | 1 | 0 | 0 | 0 | 0 | 0 | 0 | 0 | 0 | 0 |
| Sorarù et al. 2011 | ALS cases | 477 | 3 | 0 | 1 | 6 | 0 | 0 | 2 | 1 | 4 | 0 | 0 | 0 | 0 | 0 | 0 | 0 |
|  | Controls | 506 | 1 | 0 | 0 | 2 | 1 | 0 | 0 | 2 | 0 | 0 | 0 | 0 | 0 | 0 | 0 | 0 |
| Van Damme et al. 2011 | ALS cases | 3606 | 10 | 3 | 0 | 43 | 0 | 9 | 5 | 4 | 5 | 2 | 1 | 0 | 1 | 0 | 1 | 0 |
|  | Controls | 3905 | 13 | 1 | 0 | 68 | 1 | 7 | 4 | 5 | 0 | 0 | 0 | 0 | 0 | 0 | 0 | 0 |
| van Langenhove et al. 2012 | ALS cases | 135 | 2 | 0 | 0 | 3 | 0 | 1 | 2 | 0 | 0 | 1 | 0 | 0 | 0 | 0 | 0 | 0 |
|  | Controls | 1593 | 1 | 0 | 1 | 20 | 0 | 3 | 1 | 1 | 0 | 0 | 0 | 0 | 0 | 0 | 0 | 0 |
|  |  |  |  |  |  |  |  |  |  |  |  |  |  |  |  |  |  |  |

The table reports the pooled counts of CAG trinucleotide repeat alleles < 23 and counts of each allele with 24 repeats or greater of the British and Dutch

unpublished cohorts (*), and of all case-control datasets passing our inclusion criteria published after 1 August 2010, the date of the first association reported with ALS.

**Supplementary Table 4: Summary of the primary analysis conducted on fifteen low bias-risk case-control studies using a fixed effects model.**

| **Number of CAG**  **repeats** | **Relative Risk**  **(95% CI)** | **I^2^ (*p*-value of heterogeneity)** | **Reletive Risk**  ***p*-value** | **N. of intermediate**  **repeat alleles**  **(ALS group)** | **N. of Intermediate**  **repeat alleles**  **(Control group)** | **Studies with no**  **zero observations** |
| --- | --- | --- | --- | --- | --- | --- |
| 24 | 1.14 (0.84-1.55) | 55.7% (0.01) | 0.396 | 76 | 106 | 1-15 |
| 25 | 0.48 (0.31-0.74) † | 0% (0.82) | 0.001 | 30 | 73 | 1, 3-14 |
| 26 | 0.62 (0.30-1.31) † | 0% (0.78) | 0.214 | 10 | 24 | 5-6, 9-13, 15 |
| 27 | 0.82 (0.68-0.98) † | 19.7% (0.23) | 0.033 | 202 | 395 | 1-15 |
| 28 | 0.75 (0.39-1.45) † | 0% (0.84) | 0.394 | 10 | 22 | 2-3, 5-14 |
| 29 | 1.68 (1.11-2.54) † | 0% (0.61) | 0.01 | 59 | 60 | 1-15 |
| 30 | 2.02 (1.30-3.15) | 48.8% (0.02) | 0.002 | 50 | 26 | 1-7, 9-15 |
| 31 | 2.96 (1.73-5.05) † | 0% (0.56) | 8 x 10-5 | 55 | 20 | 1-12, 14-15 |
| 32 | 8.37 (4.02-17.43) † | 0% (1.0) | 3 x 10-8 | 61 | 6 | 1-10, 12-14 |
| 33 | 4.73 (1.92-11.63) † | 0% (1.0) | 0.0007 | 20 | 1 | 1-2, 4-6, 9-10, 12-15 |
| 34 | 1.79 (0.51-6.35) † | 0% (1.0) | 0.365 | 6 | 1 | 1, 5, 7, 9-10, 14 |

The count for each allele of between 24 and 34 CAG trinucleotide repeats was compared to the pooled count of alleles with ≤ 23 trinucleotide repeats (21420 for the ALS group; 30314 for the control group). The I^2^ and the Q-test *p*-value of heterogeneity were used as measures of heterogeneity between studies. A fixed effects model was used. Included studies: 1) Conforti et al. 2012; 2) Corrado et al. 2011; 3) Daoud et al. 2012; 4) Dataset-NL; 5) Dataset-UK; 6) Elden et al. 2010; 7) Gispert et al. 2012; 8) Lahut et al. 2012; 9) Lattante et al. 2014; 10) Liu et al. 2013; 11) Lu et al. 2014; 12) Ross et al. 2011; 13) Soong et al. 2014; 14) Van Damme et al. 2011; 15) Van Langenhove et al. 2012.

**Supplementary Table 5: Summary of the primary analysis conducted on fifteen low bias-risk case-control studies using a random effects model.**

| **Number of CAG**  **repeats** | **Relative Risk**  **(95% CI)** | **I^2^ (*p*-value of heterogeneity)** | **Reletive Risk**  ***p*-value** | **N. of intermediate**  **repeat alleles**  **(ALS group)** | **N. of Intermediate**  **repeat alleles**  **(Control group)** | **N. of studies with no**  **zero observations** |
| --- | --- | --- | --- | --- | --- | --- |
| 24 | 1.22 (0.69-2.15) § | 55.7% (0.01) | 0.492 | 76 | 106 | 1-15 |
| 25 | 0.50 (0.31-0.78) | 0% (0.82) | 0.003 | 30 | 73 | 1, 3-14 |
| 26 | 0.64 (0.29-1.42) | 0% (0.78) | 0.278 | 10 | 24 | 5-6, 9-13, 15 |
| 27 | 0.83 (0.66-1.04) | 19.7% (0.23) | 0.106 | 202 | 395 | 1-15 |
| 28 | 0.78 (0.37-1.58) | 0% (0.84) | 0.470 | 10 | 22 | 2-3, 5-14 |
| 29 | 1.61 (1.03-2.49) | 0% (0.61) | 0.034 | 59 | 60 | 1-15 |
| 30 | 2.58 (1.21-5.52) § | 48.8% (0.02) | 0.02 | 50 | 26 | 1-7, 9-15 |
| 31 | 2.69 (1.52-4.76) | 0% (0.56) | 0.001 | 55 | 20 | 1-12, 14-15 |
| 32 | 8.08 (4.03-16.18) | 0% (1.0) | 4 x 10-9 | 61 | 6 | 1-10, 12-14 |
| 33 | 5.10 (2.02-12.84) | 0% (1.0) | 0.001 | 20 | 1 | 1-2, 4-6, 9-10, 12-15 |
| 34 | 1.76 (0.48-6.41) | 0% (1.0) | 0.394 | 6 | 1 | 1, 5, 7, 9-10, 14 |

The count for each allele of between 24 and 34 CAG trinucleotide repeats was compared to the pooled count of alleles with ≤ 23 trinucleotide repeats (21420 for the ALS group; 30314 for the control group). The I^2^ and the Q-test *p*-value of heterogeneity were used as measures of heterogeneity between studies. A fixed effects model was used. Included studies: 1) Conforti et al. 2012; 2) Corrado et al. 2011; 3) Daoud et al. 2012; 4) Dataset-NL; 5) Dataset-UK; 6) Elden et al. 2010; 7) Gispert et al. 2012; 8) Lahut et al. 2012; 9) Lattante et al. 2014; 10) Liu et al. 2013; 11) Lu et al. 2014; 12) Ross et al. 2011; 13) Soong et al. 2014; 14) Van Damme et al. 2011; 15) Van Langenhove et al. 2012.

**Supplementary Table 6: Goodness of fit between linear and exponential models.**

|  | ***Linear model***  ***(y = x)*** | ***Exponential model***  ***(y = e^(a + b*x)^)*** |
| --- | --- | --- |
| AIC | 39.33 | 22.89 |
| BIC | 39.92 | 23.48 |

We compared the goodness of fit of linear and exponential regression models using the Akaike information criteria (AIC) and the Bayesian information criteria (BIC). The difference between models using either criterion is greater than 10, and the smaller value is for the exponential model in each case, suggesting that this is the better fit.

**Supplementary Table 7: Demographic and clinical characteristics of ALS patients using a threshold of 29 repeats.**

**Table 7a**

| **Dataset-UK** |  |  |  |
| --- | --- | --- | --- |
| **Factor** | **<29** | **≥29** | ***p*-value** |
| **Age at Onset (years), mean (SD)** | 60.82 (10.94) | 60.54 (10.95) | 0.94 |
| **Female, n (%)** | 360 (38.1) | 11 (42.3) | 0.69 |
| **Bulbar onset, n(%)** | 241 (28.2) | 4 (16) | 0.26 |

**Table 7b**

| **Dataset-NL** |  |  |  |
| --- | --- | --- | --- |
| **Factor** | **<29** | **≥29** | ***p*-value** |
| **Age at Onset (years), mean (SD)** | 60.89 (11.63) | 60.21 (12.39) | 0.77 |
| **Female, n (%)** | 522 (39.9) | 10 (52.6) | 0.35 |
| **Bulbar onset, n(%)** | 423 (32.6) | 6 (31.6) | 1.00 |

The threshold of 29 repeats was based on the meta-analysis results in Supplementary Tables 4 and 5.

a) UK data. Age at onset and gender were available for 971 patients, site of onset for 878 patients.

b) Dutch data. Age at onset was available for 1325 patients, gender for 1315, and site of onset for 1316.

**Supplementary Fig 1. Numbers of ALS cases and controls of each study included in the meta-analysis.**

**
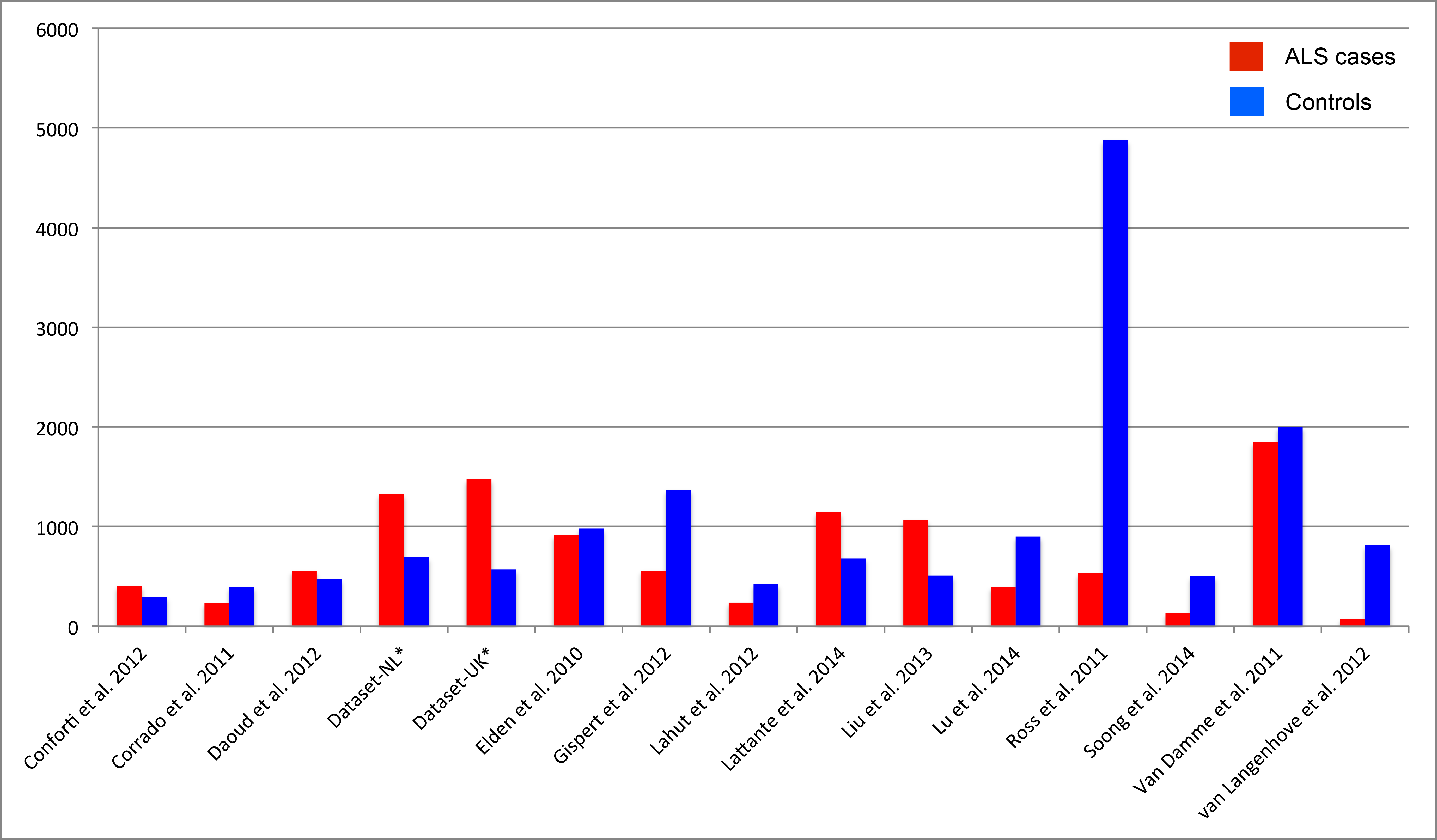
**

The bar plot reports the number of cases and controls included in each of the 15 studies used in the meta-analysis and selected

according to the Newcastle-Ottawa Scale (NOS) questionnaire for Quality Assessment of Nonrandomized Studies. (*) Unpublished data.
